# Expression and Function of BMP and Activin Membrane-Bound Inhibitor (BAMBI) in Chronic Liver Diseases and Hepatocellular Carcinoma

**DOI:** 10.3390/ijms24043473

**Published:** 2023-02-09

**Authors:** Florian Weber, Oliver Treeck, Patricia Mester, Christa Buechler

**Affiliations:** 1 Institute of Pathology, University of Regensburg, 93042 Regensburg, Germany; 2 Department of Gynaecology and Obstetrics, University Medical Center Regensburg, 93053 Regensburg, Germany; 3 Department of Internal Medicine I, Regensburg University Hospital, 93053 Regensburg, Germany

**Keywords:** liver fibrosis, immune cells, hepatic stellate cells, β-catenin, bone morphogenetic proteins

## Abstract

BAMBI (bone morphogenetic protein and activin membrane-bound inhibitor) is a transmembrane pseudoreceptor structurally related to transforming growth factor (TGF)-β type 1 receptors (TGF-β1Rs). BAMBI lacks a kinase domain and functions as a TGF-β1R antagonist. Essential processes such as cell differentiation and proliferation are regulated by TGF-β1R signaling. TGF-β is the best-studied ligand of TGF-βRs and has an eminent role in inflammation and fibrogenesis. Liver fibrosis is the end stage of almost all chronic liver diseases, such as non-alcoholic fatty liver disease, and at the moment, there is no effective anti-fibrotic therapy available. Hepatic BAMBI is downregulated in rodent models of liver injury and in the fibrotic liver of patients, suggesting that low BAMBI has a role in liver fibrosis. Experimental evidence convincingly demonstrated that BAMBI overexpression is able to protect against liver fibrosis. Chronic liver diseases have a high risk of hepatocellular carcinoma (HCC), and BAMBI was shown to exert tumor-promoting as well as tumor-protective functions. This review article aims to summarize relevant studies on hepatic BAMBI expression and its role in chronic liver diseases and HCC.

## 1. Introduction

**Figure 1:**
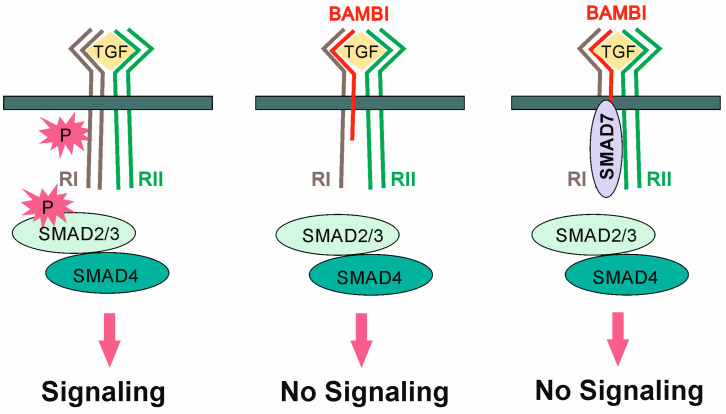
Effect of BAMBI on TGF-β signaling. Binding of TGF-β induces the formation of a complex of two TGF-βRII (RII) and two TGF-βRI (RI) molecules and phosphorylation of TGF-βRI, which subsequently phosphorylates SMAD2/3. These latter molecules associate with SMAD4; the complex enters the nucleus and regulates the transcription of target genes. BAMBI stably associates with TGF-βRI and prevents phosphorylation of this receptor. TGF-βRI and BAMBI were also shown to associate with the inhibitory SMAD7.

Transforming growth factor β receptors (TGF-βRs) comprise seven type I and five type II transmembrane receptors. Ligands such as TGF-β first bind to type II receptors with high affinity and then recruit type I receptors. By contrast, bone morphogenetic proteins (BMPs) initially bind type I receptors and then engage type II receptors. However, all ligands trigger the assembly of two type I and two type II receptors (Figure 1). The intracellular kinase domain of TGF-βRII phosphorylates and activates TGF-βRI, which then phosphorylates receptor-regulated SMAD transcription factors (R-SMADs, receptor-regulated mothers against decapentaplegic homologs), the effectors of TGF-β-induced gene regulation. TGF-β induces the phosphorylation of SMAD2/3 (Figure 1), and BMPs the phosphorylation of SMAD1/5/8. These activated R-SMADs form a complex with SMAD4, a so-called common-mediator SMAD, which enters the nucleus to regulate the transcription of TGF-β and BMP target genes [1,2,3].

A search for genes related to BMP4 signaling in Xenopus led to the identification of bone morphogenetic protein and activin membrane-bound inhibitor (BAMBI). BAMBI and BMP4 were co-expressed in Xenopus gastrulae and tadpoles, suggesting a similar expression pattern. *BAMBI* mRNA levels were induced in explants of the dorsal marginal zone from Xenopus embryos microinjected with *BMP4* mRNA, and this regulation was mediated by the BMP2/4 (activin receptor-like kinase-(ALK)3/6)) receptor [4]. BMP4 is essential for embryogenesis, and BMP4 knock-out causes embryonic lethality [5]. Yet, BAMBI knock-out mice had a mild phenotype with a range of subtle defects. These animals were viable, developed normally, and were fertile [6,7,8,9].

*NMA* (*non-metastatic gene A*), cloned in 1996, has turned out to be identical to *BAMBI* [10]. This gene was found to be highly expressed in non-metastatic melanoma cell lines, but was not detectable in highly metastatic melanoma cells and normal skin. Northern blot analysis of RNA of human organs revealed high expression of *NMA* in the spleen, kidney medulla, and placenta and low expression in the liver, gut, kidney cortex, and prostate [10]. Microarray and RNA sequencing data showed low expression of *BAMBI* in the colon, adipose tissue, and skeletal muscle, but relatively high expression in the ovary. The mRNA levels of the heart, hypothalamus, kidney, liver, lung, and testes were comparable [11].

**Figure 2:**
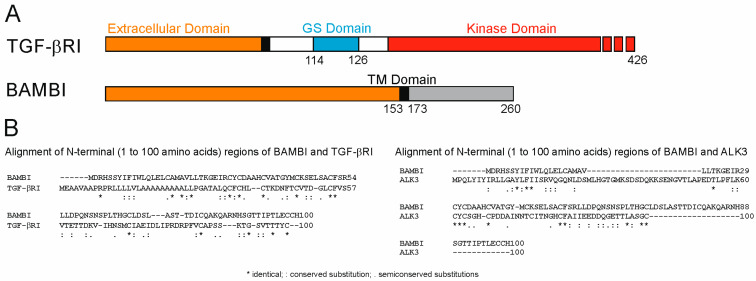
The structure of TGF-β type I receptor and BAMBI protein. (**A**) Domains of TGF-βRI and BAMBI protein (Glycine–serine-rich domain = GS domain; Transmembrane domain = TM; numbers in the figure refer to amino acid positions). (**B**) Alignment of the N-terminal 100 amino acids of TGF-βRI (NCBI Reference Sequence: NP_001124388.1) and BAMBI (NCBI Reference Sequence: NP_036474.1) as well as ALK3 (NCBI Reference Sequence: NP_001393518.1) and BAMBI using Clustal Omega.

The genomic location of human *BAMBI* is 10p12.1; the protein consists of 260 amino acids and has a molecular weight of 29 kDa [10]. BAMBI was found to structurally resemble the extracellular domain of TGF-β type I receptors containing conserved cysteine residues, and was termed pseudoreceptor because it lacks the intracellular protein kinase domain [4] (Figure 2).

BAMBI protein can associate with TGF-βRI receptors and thereby blocks the formation of the typical receptor complex, thus inhibiting the signaling of ligands such as TGF-β and BMPs [4] (Figure 1). Co-immunoprecipitation experiments found that, with the exception of ALK2, a BMP type I receptor with an important function in the development of bones and neural tissues [12], BAMBI can bind to all type I receptors, leading, for example, to inhibition of BMP4 activity by BAMBI [4]. The type II receptors TGF-βRII and activin receptor type 2 (ActR-II) were shown to also interact with BAMBI [4]. BAMBI can, moreover, form a complex with SMAD7 and TGF-βRI (Figure 1). SMAD7 is one of three inhibitory SMADs [2]. The TGF-βRI/SMAD7 complex prevents the interaction between TGF-βRI and SMAD3, and, accordingly, the activation of SMAD3 [13]. The inhibitory effects of BAMBI do not depend on its binding to the ligands TGF-β and BMP [4]. Notably, recombinant BAMBI lacking the N-terminal extracellular domain blocks the activity of endogenous BAMBI and acts as a dominant-negative inhibitor of BAMBI. This engineered protein does not bind to the TGF-βRI receptors. Wild-type and N-terminal deleted BAMBI can form a complex preventing binding of the wild-type form to TGF-βRs [4].

TGF-β, besides its role in organ fibrosis, also is a central regulator of the immune system [14]. SARS-CoV-2 (severe acute respiratory syndrome coronavirus 2) infection was shown to dysregulate TGF-β signaling and this was supposed to contribute to thrombosis and acute respiratory distress syndrome in patients [15]. Notably, Huh-7 cells with BAMBI knock-down had higher angiotensin-converting enzyme-2 mRNA and protein levels [16]. The binding of the SARS-CoV-2 spike protein to angiotensin-converting enzyme-2 (ACE2) initiates SARS-CoV-2 infection [16], indicating that BAMBI may have a role in COVID-19 and possibly the effects of this virus on the liver [17,18].

Epstein–Barr virus (EBV) infects about 90% of people worldwide, and is associated with cancers such as nasopharyngeal carcinoma and Hodgkin’s disease in some of them [19]. *BAMBI* was found overexpressed in EBV-positive compared to EBV-negative epithelial cancer cell lines. *BAMBI* mRNA levels did, however, not differ between EBV-positive and EBV-negative cancers [20], and the function of BAMBI in EBV-associated tumors needs further study.

The role of TGF-β is, however, best studied in liver fibrosis [3]. The activation of quiescent hepatic stellate cells (HSCs) by TGF-β is a key event during liver fibrosis [3]. The main insults causing hepatic injury are chronic hepatitis B virus (HBV) and hepatitis C virus (HCV) infections, ethanol abuse, and non-alcoholic fatty liver disease (NAFLD) [21,22,23,24]. A considerable portion of these patients develops progressive liver injury, characterized by hepatic inflammation and fibrosis, which may finally end in liver cirrhosis [21,22,23,24].

Obesity is a risk factor for NAFLD, but moreover exaggerates the severity of all chronic liver diseases. Liver steatosis is a relatively benign stage and is common in alcoholic and non-alcoholic liver diseases [25,26,27]. Chronic HCV infection has a higher prevalence of liver steatosis, especially in genotype 3 infected patients [28].

Excess hepatic deposition of lipids, such as free fatty acids and free cholesterol, makes the liver more susceptible to further insults. Saturated fatty acids, as is palmitate, increased TGF-β in the supernatant of primary human hepatocytes [29] and induced the expression of *TGF-*β in Huh7 liver cancer cells [30]. TGF-βRII expression and TGF-β activity were higher in HepG2 cells challenged with free cholesterol [31]. These experiments provide evidence for the upregulation of TGF-β and its receptor through lipids, and thus could connect excess hepatic deposition of specific lipid species with liver fibrosis.

The function of BAMBI as a TGF-β type I pseudoreceptor provides strong evidence for an inhibitory role in hepatic fibrosis. This review aims to summarize the current knowledge about the expression and function of BAMBI in liver diseases. Liver cirrhosis of any etiology is a risk factor for HCC [32,33], and the role of TGF-β in liver fibrosis and HCC are shortly summarized in the following paragraph.

## 2. TGF-β in Liver Fibrosis and Tumorigenesis

TGF-β signaling is a central pathway for the progression of liver diseases. TGF-β induces the expression, synthesis, and secretion of extracellular matrix (ECM) proteins. Among others, connective tissue growth factor (CTGF), collagens, fibronectin, laminin, osteopontin, biglycan, and decorin are increased. TGF-β, moreover, inhibits matrix metalloproteinase (MMPs) expression and induces their endogenous inhibitors, tissue inhibitors of metalloproteinases (TIMPs), to prevent ECM degradation. It is very well known that this cytokine activates quiescent HSCs, which in turn become myofibroblasts. These cells express alpha-smooth muscle actin (alpha-SMA) and produce ECM proteins [1,34,35].

Epithelial–mesenchymal transition (EMT) is a process in which epithelial cells temporarily lose epithelial characteristics, disassemble cell-to-cell contact structures, and acquire migration ability. Hepatocytes, HSCs, and bile duct cells can transform into myofibroblasts via EMT [36,37]. The TGF-β/SMAD2/3 signaling pathway and upregulation of the transcription factor Snail are particularly important for EMT. Snail mediates the downregulation of epithelial markers such as E-cadherin and enhances mesenchymal characteristics of the liver cells [38,39]. Thus, enhancing BAMBI expression to block TGF-β signaling seems a reasonable approach to prevent liver fibrosis.

TGF-β is also a key factor in the regulation of cell proliferation. In early HCC, TGF-β suppresses cell growth. Yet, cancer cells can surpass the antiproliferative effects of TGF-β through different strategies. In advanced tumor stages, TGF-β may even behave as a tumorigenic cytokine. In this regard, it was shown that the immune-suppressive effects of TGF-β could favor the immune evasion of the tumor cells. Using drugs that target TGF-β for HCC therapy requires a deep understanding of these signaling pathways and biomarkers to identify patients who may benefit from such therapies [34,40].

BAMBI thus protects from fibrosis but may also regulate anti-tumor immune responses and tumor growth [34,40]. There is some evidence that the pro-cancerous effects of TGF-β in the tumor cells occur at later stages of HCC, making TGF-β inhibitors attractive therapeutic options in advanced HCC [34,40].

## 3. TGF-β Family Members and Receptors in Liver Fibrosis

TGF-β activity is tightly regulated at multiple levels, and the bioavailability of further members of this protein family has a largely unexplored role herein [2]. The TGF-β family are structurally related molecules. In mammals, there are 33 TGF-β family ligands, and these include BMPs, activins, inhibins, growth differentiation factors (GDF), anti-Müllerian hormone, and left-right determination factors (LEFTYs) [2]. The secreted forms of some of these ligands are latent and require activation [41]. Depending on the cell context, latent TGF-β is activated by proteolytic cleavage within the latency-associated peptide prodomain or by a conformational change in this peptide [42]. While the role of TGF-β in liver fibrosis is clear, the function of further TGF-β family members has been less well studied. There is evidence for a profibrotic role of BMP1, BMP4, BMP8, and BMP9 [35,43]. BMP2, BMP5, BMP6, and BMP7 may be protective in this regard [35,43].

BMP6 is a main regulator of iron homeostasis and induces hepcidin expression in hepatocytes [35]. Recombinant BMP6 exerted anti-inflammatory and anti-fibrotic effects in HSCs and was protective in experimental NAFLD [44]. BMP7 overexpression in HSCs reduced the level of collagen and alpha-SMA, and protected from toxic liver fibrosis [35,45]. BMP7 interferes with TGF-β signaling and downregulates TGF-β expression [35]. Blockage of BMP9 activity improved cholestatic liver injury and carbon tetrachloride (CCL4)-induced fibrosis [35]. In hepatocytes, BMP9 signals via the type I receptors ALK1 and ALK2, the type II receptors BMPR2 and ActRII, and SMAD1/5/8 and induces expression of hepcidin, Snail, and inhibitor of differentiation (ID1) [46]. Hepcidin maintains iron homeostasis, and as an acute phase protein, is induced in acute liver injury. Chronic liver diseases are, however, mostly related to low hepcidin expression [47]. Snail is a key inducer of EMT and was found to suppress E-cadherin and to upregulate alpha-SMA in epithelial cells [48,49]. In liver cells, Snail controls EMT in concert with hepatocyte nuclear factor 4 and a number of microRNAs [50]. ID1 is a critical factor for HSC transdifferentiation to myofibroblasts. Thus, TGF-β upregulates ID1 in HSCs by the ALK1/SMAD1 pathway [51].

Activin A is produced by hepatocytes, HSCs, and liver sinusoid endothelial cells. HSCs themselves do not react to activin A but are activated by TGF-β, which is expressed in excess by activin A-exposed Kupffer cells [52]. Overexpression of LEFTY2 prevented HSCs activation and proliferation [53]. GDF11 increased the number of progenitor cells in the injured liver and protected it from fibrosis [54]. BAMBI may interfere with some of the activities of these ligands. Since the function of most of these proteins in the liver has not been analyzed in detail and the involved signaling pathways are often unknown, further experiments are required to identify the pathways activated by the different TGF-β family ligands being regulated by BAMBI.

The relatively large number of TGF-β family ligands all signal through combinations of seven type I and five type II receptors. The BMP pathway uses the type I receptors ALK1, ALK2, ALK3, and ALK6 and the type II receptors BMPRII, ActRIIA, ActRIIB, and anti-Mullerian hormone receptor (AMHR)II. The TGF-β/activin ligands activate ALK4, ALK5 (TGF-βRI), and ALK7, as well as ActRIIA, ActRIIB, and TGF-βRII [2]. The relatively small number of receptors and the large number of ligands provides a further regulatory mechanism to prevent excessive signaling, potentiate it, or switch signal transduction pathways [2].

The availability of the individual ligands, their affinities for the respective receptors, and the expression levels of the different receptors regulate the corresponding signaling pathways. In this context, it has been shown that ALK1 regulates ALK5 signaling. High levels of ALK1 in fibroblasts prevent the TGF-β/ALK5/SMAD2/3-induced upregulation of ECM protein expression, and importantly, low levels of ALK1 have the opposite effect [37]. In hepatocytes, TGF-β-induced upregulation of the ECM-associated cytokine CTGF through ALK5 was enhanced upon ALK1 knock-down [55,56].

TGF-β upregulates CTGF in hepatocytes, biliary epithelial cells, and HSCs, and CTGF is highly expressed in the fibrotic liver. CTGF enhances TGF-β bioactivity and, moreover, impairs the activity of BMP7, which can serve as a TGF-β antagonist. CTGF is essential for tissue remodeling and fibrogenesis and has an important role in the activation of HSCs [57,58].

SMADs, extracellular signal-regulated kinase (Erk)1/2, p38 mitogen-activated protein kinase (MAPK), protein kinase C, phosphoinositide 3-kinase (PI3K), and signal transducer and activator of transcription (STAT)3 all have a role in TGF-β mediated upregulation of CTGF in activated HSCs [59,60,61]. Molecules and signaling pathways, which interfere with the mutual induction of TGF-β and CTGF, are less well studied. BAMBI overexpressing lowered TGF-β mediated upregulation of CTGF in HepG2 cells and the phosphorylation of SMAD2. Whether further pathways downstream of TGF-β were impaired has not been analyzed in this study [55].

The complexity of TGF-βR signaling is a challenge for the development of therapeutic strategies to ameliorate liver fibrosis.

## 4. TGF-β Pathway in Inflammation

Persistent inflammation is an important trigger for fibrosis [62,63]. TGF-β is an anti-inflammatory cytokine and was shown to block the ‘kappa-light-chain-enhancer’ of activated B-cells nuclear factor (NF-κB) activation by Toll-like receptor (TLR)2, 4, and 5 ligands in RAW 264.7 macrophages. TGF-β induces the ubiquitination and proteasomal degradation of myeloid differentiation primary response 88 (MyD88) protein levels and thereby interferes with TLR-induced responses mediated by the adaptor molecule MyD88 [64].

Mice deficient in TGF-β suffer from organ inflammation and die as early as 3 weeks after birth [65], which is not surprising considering the anti-inflammatory role of TGF-β. Loss of TGF-βRII in mice protected against TGF-β-mediated fibrosis but enhanced NF-κB-driven inflammation [66].

There is, however, also evidence for an inflammation-promoting function of TGF-β signaling. Activation of transcription factor NF-κB by TGF-β is mediated by binding of SMAD3 to p52/RelB. TGF-β can also induce NF-κB signaling via the TGF-β-activated kinase 1 (TAK1). TAK1 activates the inhibitory κB kinase, and this enzyme phosphorylates the inhibitor of κB alpha, which becomes a target for proteasomal degradation. Removal of the inhibitor enables the NF-κB p65-p50 dimer to translocate to the nucleus and to act as a transcription factor [67,68]. TAK1 activation is a downstream effect of a variety of molecules, such as cytokines or LPS. TAK1 is essential for NF-κB activation in response to TLR ligands, interleukin 1β, and tumor necrosis factor [69]. Activation of c-jun N-terminal kinase (JNK) and p38 MAPK by TGF-β also involves TAK1 [70].

T-cells express TGF-β, and TGF-β emerged as a principal regulator of the immune system. The role of TGF-β in T cell biology has been summarized in previous review articles [14,71,72] and will not be addressed in detail herein.

**Figure 3:**
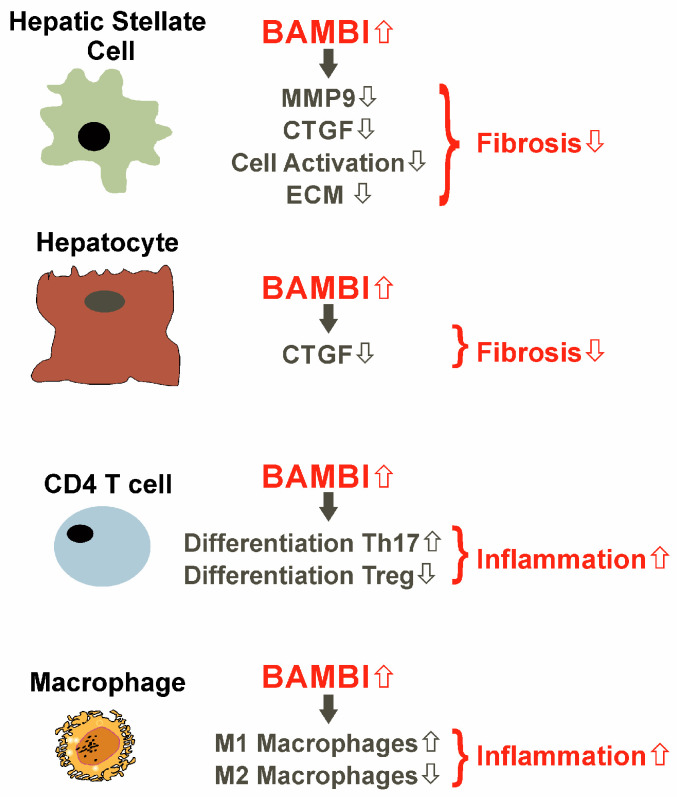
Effect of BAMBI overexpression in hepatic stellate cells (HSCs), hepatocytes, T cells and macrophages. In HSCs, BAMBI overexpression reduces cell activation, matrix metalloproteinase 9 (MMP9), connective tissue growth factor (CTGF), and extracellular matrix (ECM) protein expression. CTGF protein is also low in hepatocytes with high BAMBI levels. These effects of BAMBI protect from organ fibrosis. High BAMBI shifts T cell differentiation from regulatory (Treg) to Th17 cells and promotes the expression of M1 markers in macrophages, and both effects contribute to inflammation.

BAMBI is expressed by activated CD4 T-cells, and this is strengthened by TGF-β [73]. TGF-β, in concert with further cytokines, decides about CD4 T cells differentiation to regulatory T cells (Treg) or proinflammatory T helper type 17 (Th17) cells. BAMBI knock-out in CD4 T cells caused the cells to preferentially differentiate to Treg cells and, accordingly, reduced differentiation to proinflammatory Th17 cells when challenged with TGF-β [73] (Figure 3). M2 macrophages can induce the differentiation of Treg cells via TGF-β mediated activation of SMAD2/3. BAMBI overexpression in macrophages forces the expression of M1 markers in M2 macrophages (Figure 3) and interferes with their ability to promote differentiation of CD4 T cells to Treg cells [74].

Interestingly, an imbalance between Th17 cells and Treg cells is related to a dysregulated immune response in the liver, and may contribute to hepatic inflammation [75]. Excessive Th17 response promotes inflammation and disease progression, for example, in non-alcoholic steatohepatitis (NASH). On the other hand, Treg cells were shown to block the anti-tumoral response in cancers, and contribute to tumor growth and metastasis [75].

The function of BAMBI in immune cells has not been studied in depth, and the role of TGF-β ligands in the immune system is not further addressed in this review article.

## 5. Cell-Type Specific Expression of BAMBI in the Liver

To consider whether blockage of TGF-β activities by BAMBI overexpression is a suitable anti-fibrotic strategy, it is important to identify the liver resident cells which express *BAMBI*. Early studies suggested that BAMBI is specifically expressed in HSCs. In the mouse liver, *BAMBI* mRNA could be detected in HSCs but not in hepatocytes [76,77]. Immunohistochemistry showed co-localization of BAMBI with desmin, which is expressed by silent and activated HSCs, in the human liver [78]. Primary human HSCs were described to express BAMBI protein [55].

Later on, BAMBI protein was also detected in cell lysates of primary human hepatocytes [55]. BAMBI protein was about 12-fold more abundant in the primary human hepatocytes compared to primary human HSCs [55]. Hepatocyte cell lines are widely used in in vitro studies, and BAMBI was also detected in these cells. *BAMBI* mRNA levels were found to vary considerably between the different hepatocyte cell lines. PLC/PRF/5 cells had high and HepG2 cells undetectable expression of *BAMBI* mRNA [11]. HepG2 and Hep3B cells expressed *BAMBI* mRNA at a higher level than primary human hepatocytes. BAMBI protein was, however, hardly detectable in these cell lines [55]. Overexpression of BAMBI in HepG2 cells lowered TGF-β-induced production of CTGF protein (Figure 3) and phosphorylation of SMAD2. Interestingly, SMAD3 phosphorylation was normal [55]. This illustrates that recombinant BAMBI is functional in hepatocytes and inhibits TGF-β/SMAD2 signaling.

Cholangiocytes are the epithelial cells of bile ducts and express little BAMBI protein [55]. Neither human nor murine Kupffer cells seem to produce BAMBI mRNA [55,76]. BAMBI protein was also not detectable in primary human macrophages [55]. Expression of BAMBI in macrophages may be organ-specific because immunohistochemistry could detect BAMBI protein in alveolar macrophages [79].

BAMBI is expressed by endothelial cells [55,80]. Overexpression of BAMBI reduced, whereas knock-down increased, TGF-β-induced capillary growth and migration. In human umbilical vein endothelial cells (HUVECs), BAMBI knock-down elevated basal and TGF-β-stimulated SMAD1/5 and ERK1/2 phosphorylation, and this was prevented by BAMBI overexpression [80]. Endothelial cells from BAMBI-deficient mice had increased thrombomodulin and tissue factor pathway inhibitor protein and a higher capacity for protein C activation [7]. BAMBI null mice have a prolonged bleeding time and form unstable thrombi. Thrombin production, platelet counts, and platelet function are normal, but fibrin deposition is greatly reduced [7,8].

Fibrin deposits have been observed in different chronic liver diseases [81,82,83]. These deposits protect from biliary fibrosis but drive disease progression in NAFLD, hepatotoxic liver injury, and liver cirrhosis [81,82,83]. Regulation of fibrin deposition by BAMBI and its potential role in liver diseases is a matter of future research.

## 6. Regulation of BAMBI Expression in HSCs

**Figure 4:**
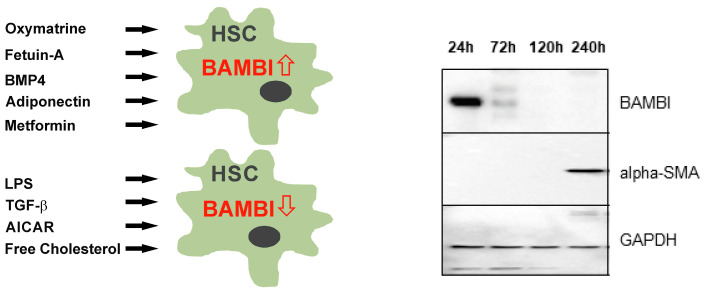
Substances regulating BAMBI expression in hepatic stellate cells (HSCs) and BAMBI expression during primary human HSC cultivation. The immunoblot shows BAMBI protein and alpha-SMA in HSCs cultivated for 24 h, 72 h, 120 h, and 240 h on plastic dishes. GAPDH is shown as loading control. 5-Aminoimidazole-4-carboxamide ribonucleotide, AICAR; bone morphogenetic protein 4, BMP4; Glycerinaldehyd-3-Phosphate-Dehydrogenase, GAPDH; Lipopolysaccharide, LPS; Transforming growth factor-β, TGF-β.

Though BAMBI is expressed by different liver resident cells, regulation of BAMBI expression was mostly investigated in HSCs. BAMBI is detectable in freshly isolated human HSCs and is downregulated during the cultivation of these cells on plastic dishes. Activated cells express alpha-SMA (Figure 4).

In chronic liver diseases, gut dysbiosis and leakage contribute to systemic and hepatic inflammation [21,84]. Activation of TLR4 by bacterial products such as lipopolysaccharide (LPS) is an important link between inflammation and fibrogenesis [85]. Surprisingly, the deletion of TLR4 in Kupffer cells could not protect from fibrosis, and TLR4 signaling in HSCs was proven to be the primary cause of liver injury [77]. Activation of murine HSCs TLR4 by LPS increased the release of several chemokines and, moreover, downregulated BAMBI [77] (Figure 4). The hepatic stellate cell line LX-2, as well as primary murine and human HSCs, had low *BAMBI* mRNA when exposed to LPS in vitro [76,86]. LPS lowered *BAMBI* mRNA levels in primary rat hepatic stellate cells but, in this study, did not change protein levels [87] (Figure 4). *BAMBI* mRNA has a half-life of about 60 min in murine glomerular endothelial cells. In these endothelial cells and in HSCs, *BAMBI* mRNA was stabilized when protein synthesis was blocked by cycloheximide, illustrating post-transcriptional regulation [88,89]. Accordingly, the 3’untranslated region of the murine and human BAMBI gene contains AU-rich elements. BAMBI protein was further shown to be degraded in endothelial cells by autophagy but not the proteasome [89]. Regulation of BAMBI mRNA and protein levels seems to be complex, and different stimuli may activate distinct regulatory pathways [88,89]. These regulatory mechanisms seem to vary between different cells types, and studies have to be performed in a cell-type-specific manner because LPS, for instance, did not lower BAMBI mRNA in glomerular endothelial cells but downregulated BAMBI expression in HSCs [76,89].

**Figure 5:**
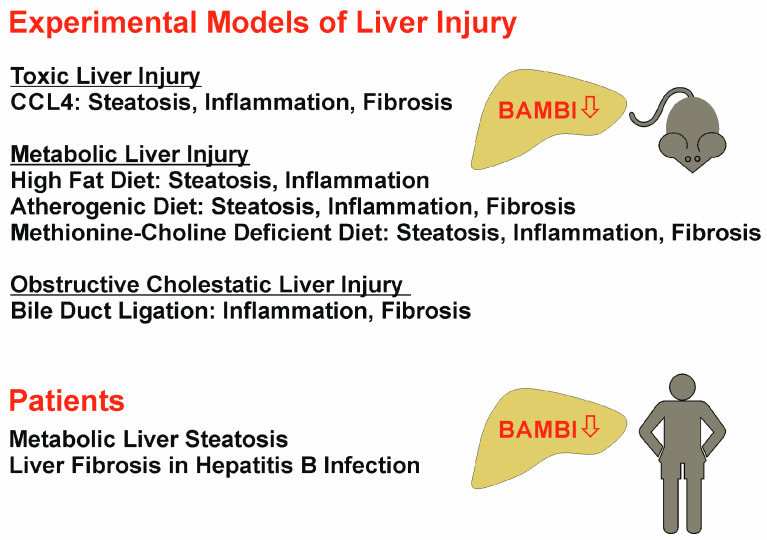
Toxic, metabolic, and obstructive cholestatic liver injury are associated with low hepatic BAMBI in murine models (carbon tetrachloride: CCL4). In patients, low BAMBI expression was described in the steatotic liver and in the fibrotic liver of patients infected with hepatitis B virus.

HSCs from LPS-injected mice indeed expressed little BAMBI [76,86]. HSCs isolated from the liver of mice with liver fibrosis expressed less BAMBI mRNA than quiescent cells [76]. Bile duct ligation causes severe liver fibrosis, and HSCs from these mice expressed little BAMBI (Figure 5). Downregulation of BAMBI did not occur in mice with mutant TLR4, illustrating a central role of TLR4 signaling for BAMBI expression in HSCs [77]. Downregulation of BAMBI by LPS involves the MyD88 NF-κB pathway [77]. The NF-κB proteins consist of p50 and p52, and the ‘Rel’ proteins p65, c-Rel, and RelB. Later on, it was shown that NF-κB p50, but not NF-κB p65, is involved in the LPS-induced downregulation of BAMBI [76].

The MyD88 NF-κB pathway is also involved in the TGF-β-induced downregulation of BAMBI protein [90] (Figure 4). TGF-β can activate NF-κB via the SMAD pathway or by activating TAK. Activated TAK initiates the proteasomal degradation of the inhibitor of κBα and, subsequently, the translocation of NF-κB p65 into the nucleus. TGF-β-induced downregulation of BAMBI protein in LX-2 cells was blocked by a MyD88 inhibitor. This blocking agent even upregulated BAMBI protein in non-stimulated LX-2 cells [90].

MicroRNA-942 induces degradation of BAMBI mRNA in HSCs. TGF-β, via ALK5 and the SMAD2/3 pathway, and LPS, via NF-κB p50, induce microRNA-942 in HSCs [78]. In line with these in vitro findings, the increased level of microRNA-942 in HBV-infected patients with F3 and F4 liver fibrosis was associated with low BAMBI mRNA and protein (Figure 5). BAMBI mRNA and protein were already about 2-fold lower in F2 than in F0/F1 fibrosis, with no difference in microRNA-942 levels suggesting the involvement of additional regulatory mechanisms [78].

Disturbed lipid metabolism was described in almost all patients with chronic liver diseases [24,28,32,91]. Free cholesterol strengthened LPS-induced downregulation of BAMBI in HSCs (Figure 4) [92]. Low expression of *BAMBI* mRNA in HSCs of mice with Niemann Pick type C disease also suggests a role for lipids as regulators of BAMBI. Niemann Pick type C is a lipid-storage disorder where cholesterol accumulates in the lysosome [93]. HSCs from NPC1-deficient mice displayed higher expression of TGF-β downstream genes when challenged with TGF-β, suggesting that besides BAMBI mRNA, its protein levels are also reduced [94].

The knock-down of BAMBI in HSCs increases TGF-β-induced collagen 1a1 and 1a2 expression. LPS and cholesterol loading had no effect on TGF-β activities when BAMBI was knocked-down, illustrating a causal role of BAMBI for cholesterol and LPS-induced HSC activation [92].

This suggests that substances that upregulate BAMBI in HSCs protect from liver injury. Fetuin-A is a glycoprotein produced by the liver and was shown to impair TGF-β signaling in LX-2 cells [95]. Recombinant fetuin-A upregulated BAMBI mRNA in the hepatic stellate cell line LX-2 and suppressed TGF-β signaling [96] (Figure 4). Whether fetuin-A protects from liver diseases is still not sufficiently explored [97]. Oxymatrine is a plant-derived alkaloid and protects against CCL4-induced liver fibrosis. This compound increased BAMBI expression in HSCs and thereby inhibited activation of these cells by TGF-β [98] (Figure 4).

Taken together, there is strong evidence for HSC-expressed BAMBI as a central molecule in liver fibrosis.

## 7. Regulation of BAMBI by Adiponectin and Metformin

Metformin is a commonly prescribed drug to improve insulin sensitivity and was shown to block TGF-β-induced activation of myofibroblasts [99,100]. Adiponectin is an adipocyte-produced protein with antidiabetic, anti-inflammatory, and hepatoprotective functions [26]. Notably, the antidiabetic drug metformin and recombinant adiponectin-induced BAMBI protein in primary human hepatocytes. Adiponectin, moreover, upregulated BAMBI protein in primary human HSCs [55] (Figure 4). In rat HSCs, an upregulation of *BAMBI* mRNA by metformin has also been demonstrated [88] (Figure 4). Adiponectin, metformin, and 5-aminoimidazole-4-carboxamide ribonucleotide (AICAR) can induce the phosphorylation of activators of 5’-AMP-activated protein kinase (AMPK). Phosphorylated AMPK stimulates catabolic pathways and switches off ATP-consuming processes [101]. Unexpectedly, AICAR lowered BAMBI mRNA in HSCs [88] (Figure 4), suggesting that AMPK activation did not mediate BAMBI upregulation. Accordingly, adiponectin induction of BAMBI in hepatocytes was not significantly blocked by the AMPK antagonist compound C [55]. Studies analyzing the relevance of these in vitro observations in patients are needed to establish a role for metformin and adiponectin in hepatic BAMBI expression.

## 8. BAMBI Expression in Non-Alcoholic Fatty Liver Disease

Convincing evidence showed that adiponectin protects from insulin resistance and non-alcoholic fatty liver disease (NAFLD) [26,102,103,104]. NAFLD is more prevalent in overweight/obese individuals and is closely linked with metabolic dysfunction. Liver steatosis is a relatively benign stage of NAFLD but sensitizes this organ to further insults. Non-alcoholic steatohepatitis (NASH) is characterized by hepatic inflammation, which can progress to liver fibrosis, cirrhosis, and HCC. Whether BAMBI is already regulated in liver steatosis has not been sufficiently studied so far. One study reported BAMBI protein to be low in human liver steatosis [55] (Figure 5). BAMBI protein expressed in the liver negatively correlated with the body mass index [55], suggesting that body weight-related factors such as adiponectin, whose circulating levels are low in NAFLD patients [102,104], may regulate hepatic BAMBI levels.

Mice fed an atherogenic diet or a methionine-choline deficient (MCD) diet develop NASH [105]. The atherogenic diet is rich in cholesterol and supplemented with cholic acid, and promotes hepatic steatosis, inflammation, and fibrosis. The atherogenic chow does not greatly change body weight [106,107,108]. The MCD diet has similar effects on the liver as the atherogenic diet but is associated with rapid body weight loss [105]. BAMBI protein was reduced in the liver of both animal models in comparison to the respective controls [55] (Figure 5). *BAMBI* mRNA was, however, not altered in the liver of MCD diet-fed mice [109].

HSCs isolated from the liver of MCD diet, high-fat diet, and high-cholesterol diet-fed mice had lower *BAMBI* mRNA than the corresponding controls [77]. Excess free cholesterol increased TLR4 protein expression in HSCs, enhanced their response to TLR4 ligands, and strengthened LPS-induced suppression of BAMBI. Free cholesterol could also lower BAMBI in the absence of LPS, but the underlying pathways have not been described [92] (Figure 4).

In a high-fat diet NASH model, hepatic downregulation of BAMBI protein was demonstrated by immunohistochemistry [87] (Figure 5). Hepatic *BAMBI* mRNA was, however, not changed in the liver of mice fed a high-fat diet. Weight loss and voluntary wheel running improved metabolic health and strongly increased hepatic *BAMBI* mRNA expression. At the protein level, BAMBI protein tended to be higher in the steatotic liver and declined in mice upon weight loss or weight loss combined with voluntary wheel running [110]. As already explained earlier, *BAMBI* mRNA expression cannot predict protein levels, and more studies analyzing BAMBI protein are needed.

The plant-derived substance sparstolonin is a TLR4 antagonist and was shown to improve oxidative stress and immune cell function in experimental NAFLD. This substance upregulated hepatic BAMBI protein [87]. Elevation of BAMBI by the probiotic substance VSL#3 may contribute to its anti-fibrotic effects in experimental NASH. Notably, this substance has no effect on inflammation [109], indicating that BAMBI interferes with TGF-β-induced fibrosis but not the anti-inflammatory effects of this cytokine.

Pioglitazone is an agonist of peroxisome proliferator receptor γ (PPARγ) and ameliorates liver inflammation and fibrosis in experimental models. In experimental NASH, TGF-β mRNA and protein were induced, and BAMBI mRNA and protein were reduced in the liver. Pioglitazone therapy prevented the NASH-associated decline of BAMBI mRNA and protein, which were aggravated by the PPARγ antagonist GW9662 [111]. It has to be noted that pioglitazone increases circulating adiponectin levels in rodents and patients [112].

BAMBI protein was, however, upregulated in the liver of rodents fed a cholesterol-rich diet [113]. Ethanol feeding alone did not change hepatic BAMBI protein but abrogated the dietary effect on hepatic BAMBI expression [113].

In summary, there is plenty of evidence for BAMBI downregulation in NAFLD. Low BAMBI levels have been detected in HSCs isolated from the injured liver (Figure 5). Whether BAMBI protein expressed in hepatocytes and/or T cells is also suppressed is currently unknown. Adipose tissue dysfunction is a key factor in the pathogenesis of NAFLD, and adiponectin, which was shown to upregulate hepatocyte and HSC BAMBI expression, is low in obesity and thus provides a link between obesity and liver fibrosis [26,114].

## 9. BAMBI Is Expressed in Adipocytes

Adipose tissue is a highly active endocrine organ secreting numerous proteins [115]. Impaired adipose tissue function in obesity is associated with low-grade systemic inflammation, dyslipidemia, and insulin resistance. Obesity is a risk factor for NAFLD and, moreover, contributes to the progression of liver diseases of any etiology [26,114,116,117,118]. The risks of developing HCC and HCC-related death are independently associated with obesity [119].

**Figure 6:**
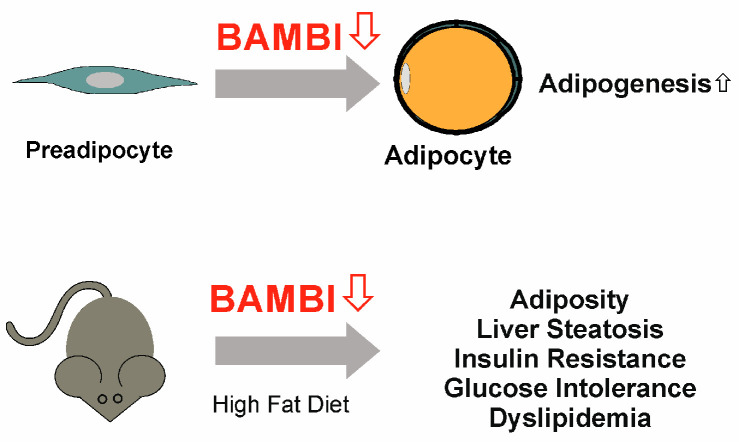
BAMBI down-regulation in adipocytes promotes adipogenesis and is associated with adiposity and metabolic disease.

BAMBI is expressed by adipocytes and is downregulated during adipogenesis. BAMBI knock-down in these cells improved adipogenesis, and increased the expression of adipocyte-specific proteins such as adiponectin. Notably, insulin-induced glucose uptake of adipocytes expressing low levels of BAMBI was markedly increased [120]. TGF-β inhibits adipogenesis, and unexpectedly, BAMBI knock-down antagonized the antiadipogenic activities of this cytokine [120,121]. On the other hand, the proadipogenic effects of BMP4 were blocked by BAMBI knock-down [120,122]. Within this context, disruption of BAMBI in adipocytes promoted obesity in high-fat diet-fed mice (Figure 6). BAMBI knock-out increased NADPH oxidase 4 and thus reactive oxygen species levels, which promoted adipogenesis.

The BAMBI null mice had liver steatosis and were glucose-intolerant, insulin-resistant, and hypercholesterolemic [123] (Figure 6). Under chow diet feeding, there were no significant differences in body weight, liver weight, subcutaneous adipose tissue, and visceral adipose tissue weights between the mutant and the control mice [123]. An about 10% lower body weight of female BAMBI null mice than the respective wild-type mice was observed in a different study [6]. Mice fed a high-fat diet and leptin-deficient obese mice had less BAMBI mRNA in subcutaneous fat than the respective control mice [120]. Body fat gain and metabolic disease may be side effects of BAMBI blockage, making the development of BAMBI-elevating drugs an attractive approach.

## 10. BAMBI and the Wnt/β-Catenin Pathway

Wingless/integrase-1 (Wnt) signaling pathways are initiated by the binding of Wnt ligands to the receptor, Frizzled (Fzd), and the co-receptors, low-density lipoprotein receptor-related proteins 5 and 6 (LRP5/6). Blockage of components of this pathway may be effective for stopping the progression of liver fibrosis or cancer. However, there is also evidence that this pathway maintains HSCs in a quiescent state, suggesting anti-fibrotic activities as well [124]. The Wnt pathway, moreover, contributes to obesity and the expansion of white adipose tissues [125].

In the absence of Wnt ligands, β-catenin is phosphorylated by glycogen synthase kinase 3 (GSK-3) and casein kinase 1, which triggers its ubiquitination and proteasome-mediated degradation. Besides GSK-3 and casein kinase 1, axin and adenomatosis polyposis coli (APC) proteins are involved herein. The binding of Wnt ligands to the receptor complex results in the recruitment of dishevelled (Dvl) to the plasma membrane, inactivation of the destruction complex, and stabilization of β-catenin. Nuclear translocation of β-catenin activates the expression of Wnt target genes in collaboration with transcription factors of the lymphoid enhancer-binding factor/T-cell factor (TCF) family. Genes regulated by β-catenin are involved in cell proliferation, migration, and invasion [126,127]. In adipocytes, serum amyloid A3, which finally promotes the proliferation of preadipocytes, was induced [125].

BAMBI is one of the targets of β-catenin/TCF4-initiated transcription and is upregulated by this pathway. Inhibitors of β-catenin/TCF and N-terminal deleted TCF4 block β-catenin-associated signaling, and both inhibitors lowered BAMBI mRNA and protein expression in human colon adenocarcinoma cells. On the other hand, COS-1 cells (African green monkey kidney cell line) expressing a degradation-resistant β-catenin protein had increased BAMBI mRNA levels [128].

**Figure 7:**
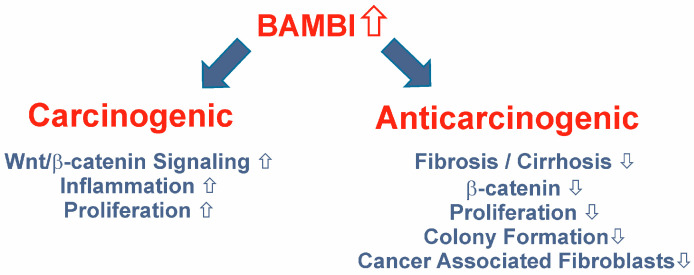
Tumor-promoting and tumor-protective functions of BAMBI overexpression.

BAMBI is upregulated by Wnt signaling pathways and further enhances the activity of this pathway (Figure 7). Overexpression of BAMBI increased the expression of Wnt target genes, and knock-down of BAMBI had the opposite effect. BAMBI can form a complex with Frizzled 5, LRP6, and Dvl2, increasing the nuclear entry of β-catenin and thereby promoting cell proliferation [129].

Overexpression of BAMBI in primary rat HSCs enhanced β-catenin gene expression, stabilized β-catenin protein, and increased cell survival. BAMBI knock-down led to lower β-catenin and induced death of these cells. Cultivation of purified HSCs on plastic dishes activates these cells [130], showing that forced overexpression of BAMBI in activated HSCs may also exert fibrosis-enhancing activities by increasing the survival of these cells. A β-catenin-dependent luciferase reporter was strongly activated by BAMBI overexpression, confirming a direct function of BAMBI in the Wnt pathway [88].

Knock-down of BAMBI in porcine preadipocytes caused β-catenin to decline and thus blocked Wnt/β-catenin signaling. BAMBI overexpression promoted the nuclear translocation of β-catenin and thereby suppressed adipogenesis [131].

There is ample of evidence that BAMBI is upregulated by the Wnt pathway which in turn is activated by BAMBI. The Wnt pathway is activated in chronic liver injury and contributes to inflammation, the proliferation of HSCs, and fibrosis [124]. BAMBI protein is, however, low in almost all experimental models of hepatic injury (Figure 5), and this suggests that pathways contributing to BAMBI suppression are much more relevant in vivo.

## 11. BAMBI Expression in HCC

The Wnt/β-catenin signaling pathway is a tumor-promoting factor in different cancers and in HCC [132]. HCCs are highly heterogeneous tumors and account for 85% of all hepatic cancers [133]. BAMBI overexpression blocks TGF-β activity, which otherwise induces cell growth arrest [128] (Figure 7). Thus, in cancer cells, high BAMBI expression is supposed to enhance tumor growth [128].

MET, AKT, and β-catenin are among the oncoproteins dysregulated in HCC [134,135,136]. Activated AKT and β-catenin, as well as activated AKT/MET, accordingly, can induce liver tumors in mice. BAMBI expression was, however, not changed in the tumors of murine models with activated MET/AKT or activated AKT/b-catenin [137], although other studies have shown that transcription of BAMBI is activated by β-catenin [128,138].

A few studies analyzed BAMBI mRNA in human HCCs. One of these analyses included five HCC cases, and BAMBI expression was increased in the tumors of three cases and comparable between tumor and non-tumor tissues of two cases [128]. BAMBI mRNA expression was also upregulated in the HCC tissues of 20 patients when compared to the respective non-tumor tissues [139]. In line with these data, human protein atlas experimental findings indicate that high expression of BAMBI mRNA in liver cancer is associated with shorter survival [140].

Autophagy can promote cancer growth by supplying nutrients to the tumor cells [141,142]. Of the 106 identified genes with a role in autophagy, which differed between HCC and normal liver, subsequent Cox regression analysis verified 10. The gene-expression signature of these ten autophagy-related genes, with one of them being BAMBI, was positively associated with an advanced tumor stage. This gene signature predicted the prognosis of patients with a 1-year area under the curve (AUC) of 0.688 and a 3-year AUC of 0.674 [143].

Further evidence for a tumor-promoting role of BAMBI came from the high expression of BAMBI in tumors with overexpression of the hepatic stem cell marker, epithelial cell adhesion molecule (EpCAM). These tumors are poorly differentiated, and are associated with a shorter overall and disease-free survival [144]. EpCAM-expressing tumors are characterized by the activation of the Wnt/β-catenin pathway and increased BAMBI [145].

*BAMBI* mRNA was also induced in Hep3B and Huh7 cells by microRNA-HCC2, which is overexpressed in HCC tissues compared to non-tumor tissues [139]. High expression of BAMBI increased cell viability and colony formation of these cell lines [139].

*BAMBI* was one of the 122 genes identified to be increased in HCC tissues with high mutation load, and was found to be closely related to prognosis [146].

In HCC tissues of HBV-positive and HBV-negative patients, the tumor to non-tumor BAMBI mRNA ratio was 1.5 and 1.4 (*p* > 0.05), respectively, showing that BAMBI expression did not greatly differ between the tumor and the non-tumor tissues [147].

Evidence for a tumor-protective function of BAMBI was obtained by a study showing that C-terminal truncated HBV X, which contributes to carcinogenesis in HBV infection, downregulated BAMBI. Overexpression of BAMBI in HepG2 cells suppressed β-catenin and TGF-β expression, and lowered cell proliferation and colony formation in soft agar (Figure 7). Xenotransplantation of the BAMBI-expressing HepG2 cells confirmed a tumor-suppressive function of this protein [138].

BAMBI expression is regulated by transcriptional, post-transcriptional, and post-translational mechanisms [88,89]. Protein levels cannot be predicted from mRNA levels, and this has been demonstrated for various other genes before [148]. As far as we know, there is only one study that has analyzed BAMBI protein in HCC. Here, BAMBI protein was strongly suppressed in HCC tissues compared to tumor-adjacent tissues of the four different patients analyzed. HepG2, Hep3B, and Huh7 cells expressed *BAMBI* mRNA, as did primary human hepatocytes. BAMBI protein was, however, hardly detectable in these cell lines [55], further illustrating that BAMBI mRNA levels are not sufficient to anticipate protein levels [148]. 

Compatible with low BAMBI protein in HCC tissues, tumor-protective roles of BAMBI have also been described. Overexpression of BAMBI inhibited TGF-β-induced differentiation of bone marrow-derived mesenchymal stem cells to cancer-associated fibroblasts (Figure 7). The upregulation of tumor-promoting factors CC-chemokine ligand 5 (CCL5) and C-X-C motif chemokine ligand 12 (CXCL12) was efficiently blocked by BAMBI. The Wnt/β-catenin pathway was not activated by BAMBI in this model, and axin2, which is a target gene of this pathway, was not regulated [149].

BAMBI was shown to exert tumor-promoting and cancer-protective functions (Figure 7). Most studies agree that *BAMBI* mRNA expression is elevated in cancer tissues compared to the respective non-tumorous tissues. Since the data for BAMBI protein levels in HCCs are limited, further analysis is required. Overexpression of BAMBI in the hepatocyte cell line HepG2 lowered proliferation, and had the opposite effect in Hep3B and Huh7 cells [138,139]. These are all well-differentiated HCC cell lines, which nevertheless differ in gene expression [150]. These cell lines have wild-type p53 (HepG2), non-sense p53 mutation (Hep3B), or a p53 point mutation (Huh7) [151]. TGF-β and p53 signaling are connected, and mutant p53 mostly subverts the tumor suppressor function of TGF-β [152]. This can, however, not fully explain the opposite effects of BAMBI overexpression in the different cell lines.

A dual role in tumorigenesis and disease progression has been described for the tumor microenvironment, which contributes to immune editing and immune surveillance but also facilitates tumor growth, metastasis, and the escape from immune surveillance [153]. TGF-β acts as a tumor suppressor in early HCC and contributes to disease progression at late stages, and clinical trials testing the effect of TGF-β inhibitors in advanced HCC are carried out [40].

BAMBI overexpression protects from fibrosis and cirrhosis, which is a risk factor for HCC development. Indeed, although HCC can develop within a non-cirrhotic liver, it occurs much less frequently [154,155]. These facts support that BAMBI could be a suitable approach for fibrosis therapy with a low risk for tumor formation. An exception is patients with NAFLD, where HCC can develop in the non-cirrhotic liver [154,155], and these persons may be less suitable for such therapeutic approaches.

## 12. Summary

**Figure 8:**
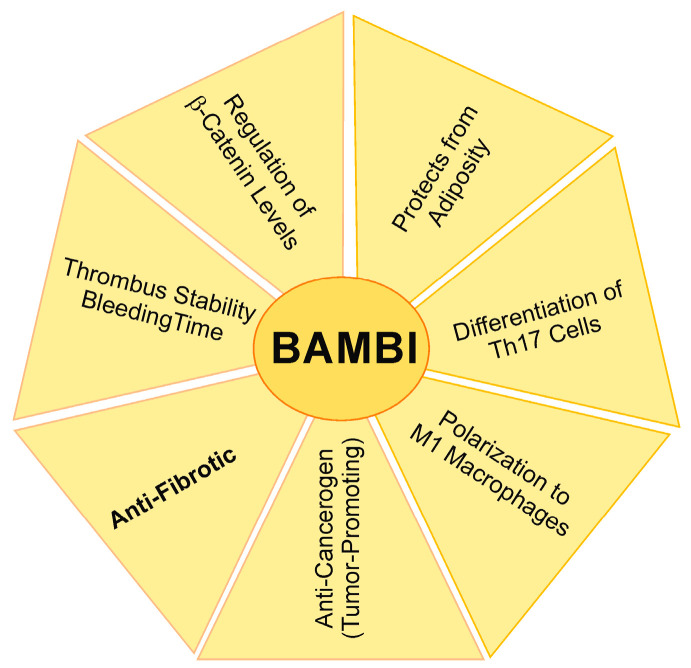
Processes regulated by BAMBI overexpression. The anti-fibrotic effects of BAMBI in the liver have been best studied so far. Whether all of the other functions of BAMBI have a role in liver diseases is unclear. Kupffer cells do, for example, not express BAMBI, and further studies are needed to clarify the effect of BAMBI overexpression in these different pathways.

BAMBI is a central molecule in liver fibrosis, and the evidence for low hepatic BAMBI levels in experimental models of liver cirrhosis is consistent. Data in the human liver are limited, and expression of hepatic BAMBI protein in HBV, HCV, and ethanol-related liver diseases has hardly been analyzed. BAMBI is expressed in HSCs, T cells, hepatocytes, cholangiocytes, and epithelial cells. The regulation and the biologic role of BAMBI have been mostly investigated in HSCs, and its function in other cell types needs further study. There is also evidence that BAMBI mRNA and protein are not coordinately changed, and thus, mRNA and protein expression should be analyzed in parallel. Experimental findings so far suggest that BAMBI overexpression protects from liver fibrosis (Figure 8), making it a promising therapeutic target. BAMBI overexpression in adipose tissues may also prevent adiposity and metabolic diseases (Figure 8). High BAMBI expression bears a risk of promoting the development of tumors, but current data suggest that the cancer-protective effects predominate (Figure 8). BAMBI, moreover, affects T cell development and their differentiation to Th17 cells and favors M1 polarization of macrophages (Figure 8), thereby contributing to hepatic and systemic inflammation. The relatively mild phenotype of BAMBI null mice suggests that loss of BAMBI is well compensated, at least as long there are no major insults. The prolonged bleeding time and formation of unstable thrombi in BAMBI-deficient mice need further attention (Figure 8). Dose effects and time points to treat liver fibrosis with this TGF-β antagonist have to be carefully evaluated in suitable studies.

In conclusion, BAMBI regulates signaling pathways involved in hepatocarcinogenesis, liver fibrosis, and inflammation. Convincing experimental evidence showed that overexpression of BAMBI protects from liver fibrosis. Inflammation triggers organ fibrosis, and the anti-fibrotic effects of BAMBI obviously outweigh the inflammatory damage upon TGF-β blockage. So far, fewer cancer-promoting than tumor-protective effects of high BAMBI have been identified. Thus, BAMBI is a versatile tool to treat liver fibrosis where no specific drug is available.
